# Azacytidine sensitizes acute myeloid leukemia cells to arsenic trioxide by up-regulating the arsenic transporter aquaglyceroporin 9

**DOI:** 10.1186/s13045-015-0143-3

**Published:** 2015-05-08

**Authors:** David Chau, Karen Ng, Thomas Sau-Yan Chan, Yuen-Yee Cheng, Bonnie Fong, Sidney Tam, Yok-Lam Kwong, Eric Tse

**Affiliations:** Division of Haematology, Department of Medicine, Queen Mary Hospital, The University of Hong Kong, Pok Fu Lam, Hong Kong, Hong Kong; Department of Pathology and Clinical Biochemistry, Queen Mary Hospital, Pok Fu Lam, Hong Kong, Hong Kong; Asbestos Diseases Research Institute, ADRI Bernie Banton Centre, University of Sydney, Concord Hospital, Sydney, Australia

**Keywords:** Acute myeloid leukemia, Arsenic trioxide, Azacytidine, Demethylating agents, Aquaglyceroporin 9

## Abstract

**Background:**

The therapeutic efficacy of arsenic trioxide (As_2_O_3_) in acute myeloid leukemia (AML) is modest, which is partly related to its limited intracellular uptake into the leukemic cells. As_2_O_3_ enters cells via the transmembrane protein aquaglyceroporin 9 (AQP9). Azacytidine, a demethylating agent that is approved for the treatment of AML, has been shown to have synergistic effect with As_2_O_3_. We tested the hypothesis that azacytidine might up-regulate AQP9 and enhances As_2_O_3_-mediated cytotoxicity in AML.

**Methods:**

Arsenic-induced cytotoxicity, the expression of AQP9, and the intracellular uptake of As_2_O_3_ were determined in AML cell lines and primary AML cells with or without azacytidine pre-treatment. The mechanism of AQP9 up-regulation was then investigated by examining the expression of transcription factors for *AQP9* gene and the methylation status of their gene promoters.

**Results:**

As_2_O_3_-induced cytotoxicity in AML cell lines was significantly enhanced after azacytidine pre-treatment as a result of AQP9 up-regulation, leading to increased arsenic uptake and hence intracellular concentration. Blocking AQP9-mediated As_2_O_3_ uptake with mercury chloride abrogated the sensitization effect of azacytidine. *AQP9* promoter does not contain CpG islands. Instead, azacytidine pre-treatment led to increased expression of HNF1A, a transcription activator of *AQP9*, through demethylation of *HNF1A* promoter. HNF1 knockdown abrogated azacytidine-induced *AQP9* up-regulation and almost completely blocked intracellular As_2_O_3_ entry, confirming that azacytidine enhanced As_2_O_3_-mediated cell death via up-regulation of HNF1A and hence increased AQP9 and As_2_O_3_ intracellular concentration. Azacytidine sensitization to As_2_O_3_ treatment was re-capitulated also in primary AML samples. Finally, azacytidine did not enhance arsenic toxicity in a liver cell line, where *HNF1A* was largely unmethylated.

**Conclusions:**

Azacytidine sensitizes AML cells to As_2_O_3_ treatment, and our results provide proof-of-principle evidence that pharmacological up-regulation of AQP9 potentially expands the therapeutic spectrum of As_2_O_3_. Further clinical trial should evaluate the efficacy of azacytidine in combination with As_2_O_3_ in the treatment of AML.

**Electronic supplementary material:**

The online version of this article (doi:10.1186/s13045-015-0143-3) contains supplementary material, which is available to authorized users.

## Introduction

Arsenic trioxide (As_2_O_3_) is an important and highly efficacious drug in the management of acute promyelocytic leukemia (APL) [1,2]. It was first successfully used as a salvage therapy for relapsed APL [3]. More recently, As_2_O_3_ has also been shown to be effective as first line induction therapy in newly diagnosed APL [4,5] and as a maintenance therapy [6]. The unique sensitivity of APL cells to As_2_O_3_ is likely to be related to As_2_O_3_-mediated degradation of the PML-RARA chimeric protein, which is an oncogenic protein produced as a result of the specific chromosomal translocation t(15;17)(q22;q12) [7,8]. In addition, As_2_O_3_ has also been shown to induce apoptosis through the generation of superoxides and reactive oxygen species [9,10], disruption of the mitochondrial transmembrane potential with release of cytochrome c and caspase activation [11], and inhibition of DNA methyltransferase DNMT leading to demethylation of tumor suppressor genes [12].

Because As_2_O_3_ must enter cells before exerting its cytotoxic activity, the control of arsenic trafficking through the plasma membrane could conceivably modulate arsenic sensitivity. Aquaglyceroporin 9 (AQP9) is a transmembrane solute transporting protein and is expressed in human leukocytes, liver, lung, and spleen [13]. Unlike the typical aquaporin water channel, it also facilitates the passage of glycerol and many other non-charged solutes. We and others have shown that AQP9 controls the transmembrane transport of As_2_O_3_, thereby playing a critical role in determining the sensitivity of cells towards As_2_O_3_-induced cytotoxicity [14,15]. In myeloid leukemia cells, expression levels of AQP9 are directly proportional to intracellular arsenic concentrations upon As_2_O_3_ treatment, which translate into increased arsenic sensitivity [14]. Of the different subtypes of acute myeloid leukemia (AML), APL cells express the highest concentration of AQP9, which might in part explain their exquisite sensitivity to As_2_O_3_ [14]. Furthermore, all-trans retinoic acid (ATRA) up-regulates AQP9 expression, contributing to its synergistic cytotoxic effect with As_2_O_3_ [14]. However, the regulation of AQP9 expression remains unclear. Understanding the mechanisms controlling AQP9 expression may enable pharmacological strategies to be designed to up-regulate AQP9 in leukemia cells, hence constituting a potential method to expand the therapeutic spectrum of As_2_O_3_ in the treatment of AML.

Demethylating agents, including azacitidine and decitabine, are a standard medication for the management of myelodysplastic syndrome and elderly subjects with AML [16-19]. Recently, demethylating agents have been shown to synergize with As_2_O_3_ in the treatment of AML *in vitro* and *in vivo* [20,21]. However, the biological basis of the synergism between demethylating agents and As_2_O_3_ has not been defined.

In this study, we proposed that one of the mechanisms of synergism between demethylating agents and As_2_O_3_ might be through modulation of AQP9 expression. To test this hypothesis, we examined the effect of azacytidine treatment on AQP9 expression and plasma membrane arsenic trafficking in AML cell lines and primary AML samples.

## Materials and methods

### Cells and reagents

The human myeloid leukemia cell lines HL-60 and K562 (purchased from ATCC, Manassas, VA, USA) and the APL cell line NB4 (a kind gift from Dr. Shen ZX, Shanghai Institute of Hematology, Rui Jin Hospital, Shanghai, China) were cultured in RPMI-1640 supplemented with 10% fetal bovine serum (FBS) and 1% penicillin/streptomycin in 5% CO_2_ at 37°C. They have been characterized and tested as described previously [14]. The human leukemia line OCI-AML3 (purchased from DSMZ, Braunschweig, Germany) was cultured in α-MEM with 20% FBS in similar conditions. The immortalized human liver cell line MIHA (a kind gift from Dr. J Roy-Chowdhury, Albert Einstein College of Medicine, New York, USA) was cultured in DMEM with 10% FBS. MIHA has been characterized and tested as described previously [22]. Primary AML samples from peripheral blood (PB) and/or bone marrow (BM) were obtained with informed consent from patients treated at Queen Mary Hospital, Hong Kong. Primary cells were cultured in StemSpan H3000 supplemented with StemSpan CC100 cytokine cocktail (StemCell Technologies, Vancouver, Canada). Archival samples were obtained from marrow mononuclear cells of AML patients stored at −80°C. Procurement of these samples was approved by the institute review board according to the Declaration of Helsinki. The demethylating drug azacytidine (5-aza-2′deoxycytidine; 5′Aza) and As_2_O_3_ were obtained from Sigma-Aldrich (St. Louis, MO, USA). The polyclonal phycoerythrin (PE)-conjugated anti-AQP9 and PE-conjugated isotypic control antibodies were purchased from Bioss Antibodies (Bioss Inc., Woburn, MA, USA).

### As_2_O_3_ cytotoxicity

Cells pre-treated with or without azacytidine (5 μM for 3 days) were washed twice with phosphate-buffered saline (PBS), re-suspended in fresh RPMI-1640 supplemented with 10% FBS, and treated with various concentration of As_2_O_3_ (0.0, 0.3125, and 0.625 μM for NB4; 0.0, 2.5, 5.0, and 10.0 μM in other cells). For experiments where AQP9 blockade was involved, cells were incubated in addition with mercury chloride (HgCl_2_) at 10 μM for 2 hours. For 3-[4,5-dimethylthiazol-2-yl]-2,5 diphenyl tetrazolium bromide (MTT) assay, 100 μL of each cell suspension was incubated for 48 hours in 96-well plates, followed by the addition of MTT reagents (10 μL for 4 hours) and the solubilizing buffer (100 μL overnight), and absorbance measurement at 560 nm. All experiments were performed in triplicates.

### Flow cytometric analysis

For apoptosis assay, cells treated with or without azacytidine were analyzed for apoptotic cells using a Cytomics FC 500 flow cytometer (Beckman Coulter, Brea, CA, USA), using an annexin V: phycoerythrin and 7-AAD apoptosis detection kit (BD Biosciences, San Jose, CA, USA). Cells were incubated with respective antibodies for 15 min and subjected to flow cytometric analysis. At least 10,000 events were collected. All flow cytometry plots and data were acquired from at least three independent experiments.

### Quantification of gene expression

Total RNA was extracted using Trizol reagent (Life technologies, Carlsbad, CA, USA), and 1 μg of RNA was reversely transcribed (SuperScript III First Strand Synthesis system, Life technologies, Carlsbad, CA, USA). The resulting cDNAs were used for semi-quantitative reverse transcription polymerase chain reaction (RT-PCR) or quantitative RT-PCR (q-RT-PCR). Primers for quantification of target and control genes were designed by the Primer Express software (Applied Biosystems, Life technologies, Carlsbad, CA, USA) (primer sequences and reaction conditions were listed in Additional file 1). Quantitative RT-PCR was performed using Power SYBR Green PCR Master Mix (Applied Biosystems, Carlsbad, CA, USA) and a StepOnePlus Real Time PCR system (Applied Biosystems, Carlsbad, CA, USA). The expressions of target genes with respect to the internal control gene were analyzed with the comparative C_T_ (ΔΔC_T_) method. Experiments were performed in triplicates.

### Western immunoblotting

Cells were washed twice with PBS and were then lysed in RIPA buffer [RIPA: 50 mM of Tris-HCl buffer pH 7.4, 150 mM NaCl, 1 mM EDTA, 1% (*v*/*v*) NP-40, and 0.25% (*w*/*v*) sodium deoxycholate, with the addition of a protease inhibitor cocktail (Roche Diagnostic, Sulzfeld, Germany), 1 mM phenylmethylsulfonyl fluoride (PMSF)]. Total protein concentration was determined using the BCA protein assay kit (Pierce Biotechnology, Rockford, IL, USA) according to the manufacturer’s instructions. For Western blot analysis, antibodies against HNF1A (Cell Signaling, Danvers, MA, USA) and β-actin (Sigma, St. Louis, MO, USA) were used. For detection of bound antibodies, HRP-conjugated secondary antibodies were used (Life Technologies (Carlsbad, CA, USA)).

### RNA interference and transfection

Gene knockdown experiments were performed with siRNA (sequences of siRNA in Additional file 1), with negative controls (AllStars Negative Controls, Qiagen, Venlo, Netherlands). K562 cells (2 × 10^5^ cells in 100 μL) were transfected with siRNA (200 ng diluted in RPMI-1640 medium, incubated at room temperature for 10 min with 6 μL HiPerfect transfection reagent, Qiagen, Germantown, MD, USA) for 6 h in 5% CO_2_ at 37°C, after which 600 μL of medium with FBS and antibiotics was added, followed by another 72 h of incubation before analysis. Experiments were performed in triplicates.

### Measurement of intracellular arsenic concentration

Cells were harvested, washed twice in ice-cold PBS, pelleted, and lysed in 0.9 mL of double-distilled water by sonication for 10 min. Yttrium (Chem Service, West Chester, PA, USA) dissolved in 2% nitric acid to ten parts per billion was then added as an internal standard to the lysate, which was vortexed and centrifuged at 3000 × *g* for 10 min. The supernatant was collected and assayed for arsenic concentration by inductively coupled plasma-mass spectrometry [23]. Experiments were performed in triplicates.

### Bisulfite modification of DNA, methylation-specific PCR and combined bisulfite restriction analysis

Genomic DNA was isolated with the QIAamp DNA Blood Mini Kit (Qiagen, Germantown, MD, USA). Bisulfite modification of DNA was performed with the Zymo DNA modification kit (Zymo Research, Irvine, CA, USA). Methylation-specific PCR (MSP) specific primers for the methylated and unmethylated alleles were designed using the OLIGO 6 primer analysis software (Molecular Biology Insights, Cascade, CO, USA) (primer sequences in Additional file 1: Supplementary Information), and the PCR products were analyzed by gel electrophoresis. CpGenome universal methylated DNA (Chemicon International Inc, Billerica, MA, USA) was used as a positive control. For combined bisulfite restriction analysis (COBRA), bisulfite-modified DNA before and after treatment was amplified by PCR with primers spanning the CG-rich region of target gene promoter (primer sequences in Additional file 1: Supplementary Information) and digested with the restriction endonuclease *Bst*UI (New England Biolabs, Ipswich, MA, USA). PCR products were analyzed by gel electrophoresis to determine the respective methylation status.

### Statistical analysis

Comparative analysis of cell viability and AQP9 expression were performed by the Student’s *t*-test, whereas correlation analysis on AQP9 and HNF1A expression was performed using the Pearson’s *χ*^2^-test. All values were presented as mean ± standard deviation (SD). Differences were considered statistically significant when *p* value was <0.05.

## Results

### Azacytidine sensitized leukemia cells to As_2_O_3_-induced cytotoxicity

**Figure 1:**
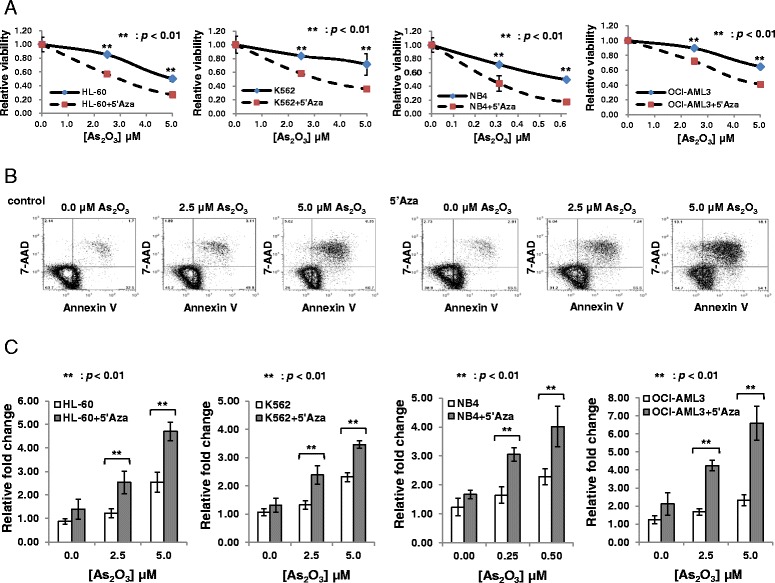
Sensitization of leukemia cells to As_2_O_3_-induced cytotoxicity with azacytidine (5′Aza) pre-treatment. **(A)** Dose-dependent cytotoxicity of As_2_O_3_ with or without 5′Aza treatment in the human AML cell lines, HL-60, K562, NB4, and OCI-AML3. As determined by MTT assay, all four cell lines pre-treated with 5′Aza exhibited significant decrease in cell viability as compared with their untreated controls. Data were acquired from three independent experiments, and relative survival of each individual cell line was normalized to its respective control. **(B)** Representative plot of flow cytometric analysis, showing a significant increase in As_2_O_3_-mediated cytotoxicity (annexin V-positive cells) in K562 cells pre-treatment with 5′Aza, as compared with control cells not treated with 5′Aza. **(C)** Bar charts showing the fold changes of As_2_O_3_-induced cell death (annexin V-positive cells) in each AML lines with or without 5′Aza pre-treatment. Quantitative analysis by flow cytometry further confirmed a significant increase in As_2_O_3_-induced apoptotic cell death after 5′Aza pre-treatment.

Of the four leukemia cell lines tested, NB4 showed the highest sensitivity to As_2_O_3_, whereas HL-60, K562, and OCI-AML3 had comparable sensitivities. Pre-treatment with azacytidine significantly sensitized these cells to As_2_O_3_-induced cytotoxicity (Figure 1A). Similar results were obtained with the annexin V/7-AAD apoptosis assay (Figure 1B,C).

### Azacytidine increased AQP9 level and enhanced intracellular entry of As_2_O_3_

**Figure 2:**
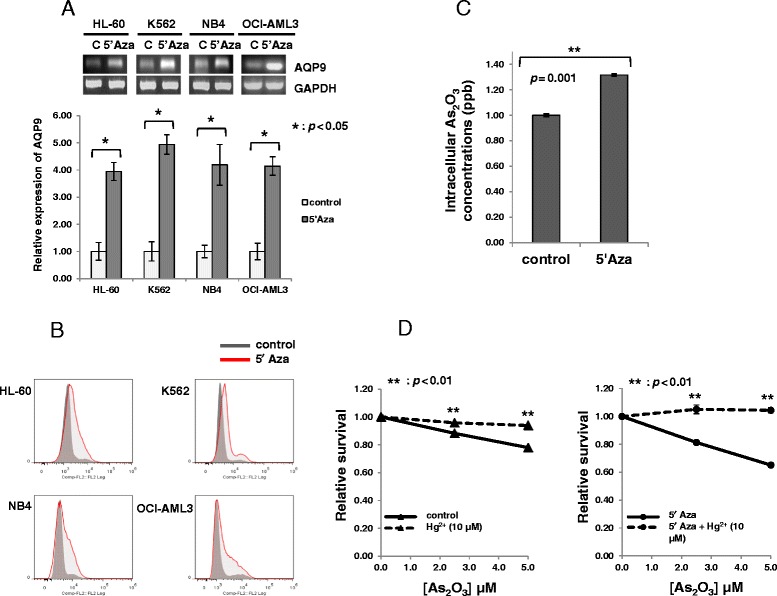
Azacytidine-mediated up-regulation of AQP9 expression and intracellular arsenic uptake. **(A)** AQP9 mRNA expression was significantly up-regulated with 5′Aza treatment in HL-60, K562, NB4, and OCI-AML3 cells, as determined by RT-PCR (upper panel) and quantitative RT-PCR (lower panel). Sample data were normalized to the control of each cell line individually by the ΔΔC_T_ method. **(B)** Up-regulation of membrane AQP9 protein expression was also confirmed by flow cytometric analysis. **(C)** Intracellular elemental arsenic concentration is significantly increased in K562 cells after pre-treatment with 5′Aza. **(D)** Treatment of K562 cells with HgCl_2_ completely abrogated the cytotoxicity induced by As_2_O_3_, either alone (left panel) or in cells pre-treatment with 5′Aza (right panel).

RT-PCR showed that azacytidine treatment significantly increased *AQP9* gene expression (Figure 2A, upper panel). Quantitative RT-PCR confirmed a four- to fivefold increase in AQP9 mRNA upon azacytidine treatment (Figure 2A, lower panel). Flow cytometric analysis showed a corresponding increase in surface expression of AQP9 (Figure 2B). The azacytidine-induced increase in AQP9 expression was functional, as it resulted in enhanced arsenic entry into cells (Figure 2C). To verify that AQP9 actually mediated increased arsenic entry into cells, which then led to cytotoxicity, the effect of AQP9 blockade with HgCl_2_ was examined [24]. As shown in Figure 2D (left panel), HgCl_2_ significantly attenuated the cytotoxicity induced by As_2_O_3_. More importantly, HgCl_2_ treatment also abolished the sensitization to As_2_O_3_ resulting from pre-treatment with azacytidine (Figure 2D, right panel). These results illustrated that azacytidine sensitized cells to As_2_O_3_ by increasing AQP9 expression, leading to increased arsenic entry and therefore enhanced cytotoxicity on subsequent exposure to As_2_O_3_.

### Transcription factor HNF1A was up-regulated by azacytidine treatment

**Figure 3:**
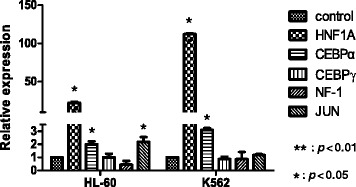
Quantitative RT-PCR analysis of the expression of potential transcription factors for the *AQP9* gene after 5′Aza treatment in HL-60 and K562 cells. The expressions of five transcription factors, including HNF1A, CEBPα, CEBPγ, NF-1, and JUN, with or without 5′Aza treatment in HL-60 and K562 cells, were determined by quantitative RT-PCR. Sample data were normalized to the control gene *GAPDH* for each cell line individually.

A thorough analysis of the DNA sequence of the *AQP9* promoter did not show any CpG islands, implying that up-regulation of AQP9 with azacytidine is not due to *AQP9* promoter demethylation. Examination of the *AQP9* promoter via the online database (UCSC Genome Browser on Human Mar. 2006 (NCBI/hg18) Assembly) and published literature showed putative DNA binding sites for several transcription factors, including hepatic nuclear factor 1A (HNF1A), CCAAT/enhancer-binding protein α (C/EBPα), C/EBPγ, c-JUN, and NF-κB [13]. Quantitative RT-PCR showed that HNF1A was the most significantly and consistently up-regulated transcription factor after azacytidine treatment (Figure 3).

### Promoter methylation of HNF1A impacted on AQP9 expression and hence transmembrane arsenic trafficking

**Figure 4:**
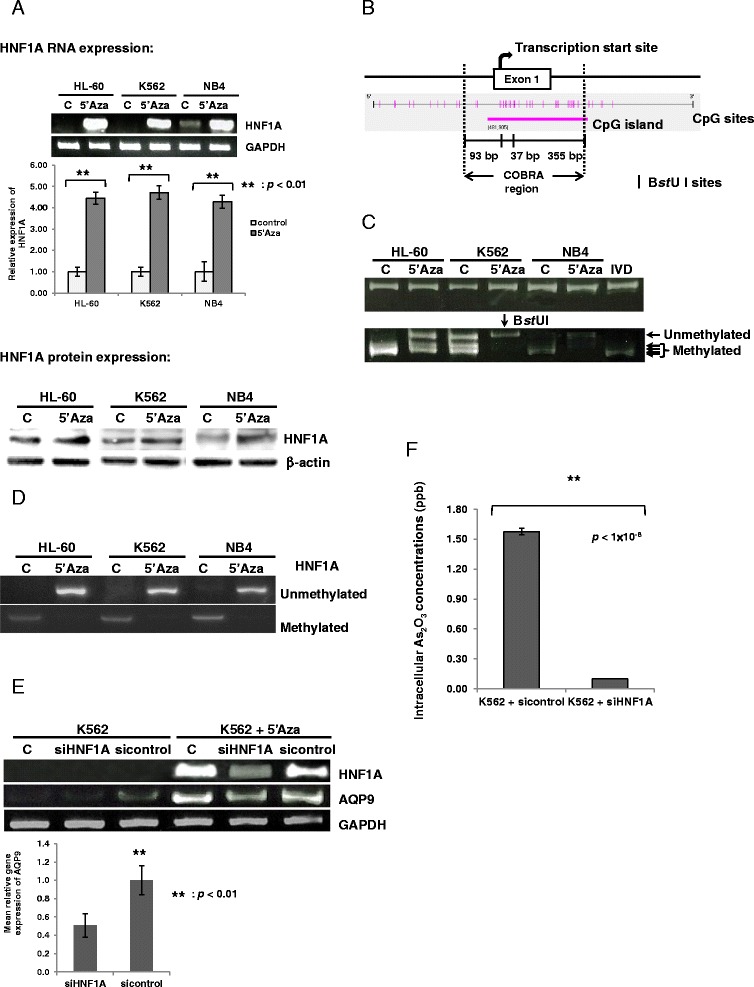
Transcription factor HNF1A was involved in azacytidine-induced up-regulation of AQP9. **(A)**. HNF1A mRNA and protein expressions were markedly up-regulated after treatment of 5′Aza as examined by semi-quantitative and quantitative RT-PCR (upper panel) and western immunoblotting (lower panel). **(B)** Diagram depicting the regions of the HNF1A promoter analyzed by Methylation-specific PCR (MSP) and Combined Bisulfite Restriction Analysis (COBRA). **(C)** The methylation status of *HNF1A* gene promoter was extensively studied and determined using combined bisulfite restriction analysis. 5′Aza treatment resulted in demethylation of the *HNF1A* gene promoter. (C: control; IVD: universal methylated DNA). **(D)** Methylation-specific PCR (MSP) was performed using PCR primers specific for methylated or unmethylated *HNF1A* gene promoter. The results showed that the *HNF1A* gene promoter was highly methylated, and 5′Aza treatment led to demethylation of the *HNF1A* gene promoter. **(E)** Specific HNF1A siRNA abrogated 5′Aza-induced AQP9 up-regulation as determined by RT-PCR (upper panel) and quantitative RT-PCR (lower panel). **(F)** In K562 cells treated with 5′Aza, specific HNF1A siRNA almost completely blocked the intracellular entry of As_2_O_3_ as compared with the control siRNA-treated cells. The results showed that HNF1A was the mediator for up-regulation of AQP9 after 5′Aza treatment.

HL-60 and K562 cells expressed very low levels of HNF1A, whereas NB4 cells had a slightly higher basal HNF1A expression (Figure 4A). Treatment with azacytidine led to a marked increase in HNF1A mRNA and protein expression in all three leukemia lines (Figure 4A). The HNF1A promoter contains a stretch of CpG islands (Figure 4B). With COBRA, the *HNF1A* promoter was found to be highly methylated in all three AML cell lines, and treatment with azacytidine led to demethylation of the *HNF1A* promoter (Figure 4C). The COBRA findings were replicated in MSP analysis (Figure 4D). To show that azacytidine treatment leading to promoter demethylation and re-expression of HNF1A was biologically relevant, siRNA was used to knock-down HNF1A. As shown in Figure 4E, K562 cells treated with azacytidine showed considerable up-regulation of HNF1A and hence AQP9, which was significantly suppressed with HNF1A siRNA. This resulted in an almost complete blockade of arsenic entry into K562 cells (Figure 4F).

### HNF1A methylation and AQP9 expression in primary AML cells

**Figure 5:**
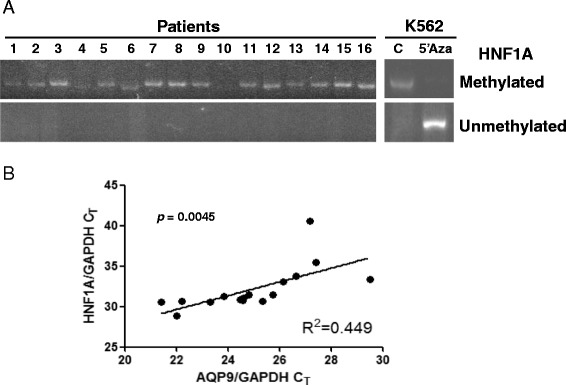
Methylation status of *HNF1A* promoter and correlation between the expressions of AQP9 and HNF1A in primary AML samples. **(A)** MSP showing highly methylated *HNF1A* promoter in 16 human AML samples. **(B)** Quantification of AQP9 and HNF1A expressions in 16 human AML samples. Result was analyzed using Pearson test. AQP9 level was found to correlate positively with HNF1A level (*p* = 0.0045).

In an archival panel of 16 non-APL AML cells, MSP showed that *HNF1A* promoter was highly methylated (Figure 5A). On quantitative RT-PCR, expressions of HNF1A and AQP9 were positively correlated, confirming that HFN1A was an important transcription activator of the *AQP9* gene in AML (Figure 5B).

### Azacytidine increased AQP9 level and enhanced As_2_O_3_-mediated cytotoxicity in primary human AML cells

**Figure 6:**
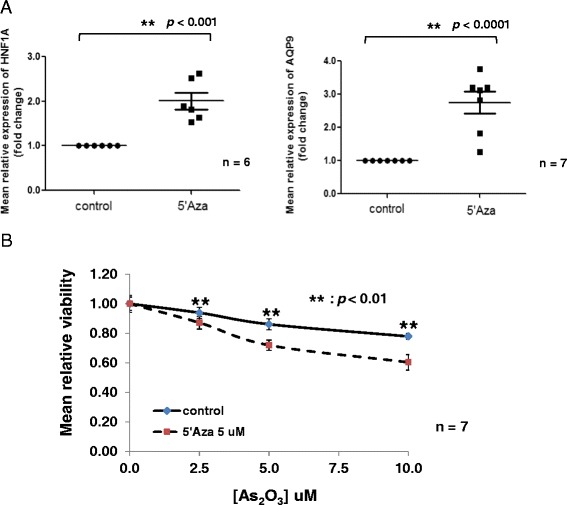
Pre-treatment with azacytidine sensitized primary AML cells to subsequent treatment with As_2_O_3_. **(A)** Treatment with 5′Aza resulted in significant up-regulation of HNF1A (left panel) and AQP9 (right panel) as determined by quantitative RT-PCR. **(B)** As_2_O_3_-mediated cytotoxicity in primary AML cells with or without 5′Aza pre-treatment as determined by MTT analysis. Sample data were normalized to the controls of each patient individually. Results represented triplicates of seven samples of primary AML cells.

Circulating AML blasts from seven patients with non-APL AML were examined. Treatment with azacytidine led to a significant increase in HNF1A and AQP9 expression (Figure 6A). Furthermore, pre-treatment with azacytidine significantly sensitized AML cells to subsequent As_2_O_3_ treatment in all cases (Figure 6B).

### Azacytidine pre-treatment and subsequent As_2_O_3_ treatment did not lead to enhanced cytotoxicity in human liver cell line

**Figure 7:**
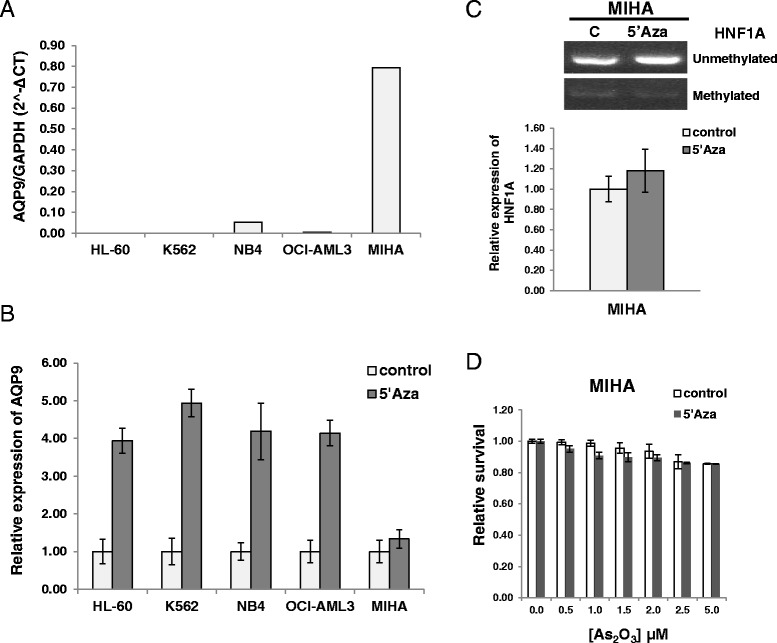
Azacytidine did not enhance hepatotoxicity of As_2_O_3_ *in vitro*. **(A)** Basal expression of AQP9 was much higher in MIHA compared with those of AML cells. **(B)** 5′Aza treatment did not increase AQP9 expression significantly in MIHA. **(C)** HNF1A promoter was hypomethylated in MIHA (upper panel), and 5′Aza treatment did not further increase HNF1A expression (lower panel). Sample data were normalized to the respective control. **(D)** Pre-treatment with 5′Aza did not significantly enhance the cytotoxicity of As_2_O_3_ in MIHA cells. Sample data were normalized to the respective control individually.

One of the major toxicities of As_2_O_3_ treatment is liver damage, so it is important to determine if azacytidine treatment might exacerbate hepatotoxicity. In the primary liver cell line MIHA [22], basal AQP9 expression was much higher than that of the AML cell lines (Figure 7A). Treatment with azacytidine did not significantly increase AQP9 expression (Figure 7B). MSP showed that the *HNF1A* promoter was almost totally unmethylated in MIHA cells, and expectedly treatment with azacytidine did not lead to a significant increase in HNF1A expression (Figure 7C). Finally, pre-treatment of MIHA cells with azacytidine did not enhance the cytotoxicity of subsequent As_2_O_3_ treatment (Figure 7D).

## Discussion

With the use of ATRA and chemotherapy, complete remission (CR) rates of over 90% can be achieved in newly diagnosed APL. In relapsed APL, As_2_O_3_ has been shown to be a highly effective salvage therapy [3]. Combination therapy with As_2_O_3_ and ATRA as frontline treatment for APL also results a very high CR rate and improved long-term outcomes [4,5]. We have also shown that the use of As_2_O_3_ and ATRA as maintenance therapy is safe and may decrease relapse in APL patients in first CR [6]. Our previous study showed that one of the mechanisms underlying the synergistic interaction between As_2_O_3_ and ATRA might be ATRA-mediated up-regulation of AQP9, resulting in increased cellular uptake and hence intracellular concentrations of arsenic [14].

In this study, we tested the hypothesis that AQP9 might be involved in the observed synergistic interaction of azacytidine with As_2_O_3_. Because we wanted to minimize the cytotoxic action of azacytidine as a confounding factor in our experiments, azacytidine was used only as a pre-treatment and removed from the culture system during subsequent As_2_O_3_ treatment. In this way, we showed that azacytidine pre-treatment sensitized cells to As_2_O_3_-induced cytotoxicity.

Our data clearly showed that azacytidine up-regulated AQP9 through demethylation of the promoter and therefore increased transcription of HNF1A, itself a transcription activator of *AQP9*. The increase in AQP9 expression at the transcription and protein levels led to increase arsenic uptake and intracellular concentrations, thereby enhancing As_2_O_3_-induced cytotoxicity. Blockade of AQP9 by HgCl_2_ and siRNA knock-down of *HNF1A* both suppressed azacytidine-induced As_2_O_3_ sensitization. Because azacytidine was removed after pre-treatment, its intrinsic cytotoxicity did not contribute to the subsequent cytotoxicity observed with As_2_O_3_ treatment. However, in actual clinical practice, if azacytidine were to be combined with As_2_O_3_ in treating patients, the inherent cytotoxicity of azacytidine might further enhance cell-kill and hence the clinical efficacy of this combination.

One concern of combined azacytidine/As_2_O_3_ treatment would be potentiation of arsenic toxicity in other organs and tissues where AQP9 is also up-regulated. The liver is a key organ that is affected by As_2_O_3_ treatment, particularly when As_2_O_3_ is used in the oral formulation due to a first-pass effect. Reassuringly, our preliminary data in MIHA showed that liver cells already expressed a high level of AQP9, which was not further increased by azacytidine treatment. Interestingly, it had been shown that As_2_O_3_-related toxicities, especially in the heart and liver, were more pronounced in *AQP9-null* mice when compared with wild-type mice, as a result of reduced As_2_O_3_ clearance [25]. Finally, in a phase I clinical trial examining As_2_O_3_ in combination with another demethylating drug decitabine in AML, no excessive adverse effects were reported [21]. Therefore, concomitant azacytidine and As_2_O_3_ treatment might not lead to increased toxicity in other organs.

The transcription regulation of *AQP9* has not been thoroughly investigated, and other pharmacological agents may also activate *AQP9* gene through various mechanisms to enhance the anti-tumor effects of As_2_O_3_ [14,15]. Previous investigations had shown that indirubin and tanshinone IIA up-regulated *AQP9* expression and augmented the anti-leukemia effect of As_2_O_3_ as present in the Realgar-Indigo naturalis formula [26]. As AQP9 transmembrane protein can be detected and quantified by fluorochrome-conjugated antibody and flow cytometric analysis, a high-throughput screening is therefore feasible in the future to identify compounds that may up-regulate AQP9 and potentially enhance the therapeutic effect of As_2_O_3_.

HNF1A is a transcription factor and is highly expressed in liver, pancreas, and kidneys [27]. It plays a critical role in transcriptional activation of differentiated hepatocyte-specific genes critical for liver function such as albumin [28]. Epigenetic regulation is involved in tissue-specific expression of HNF1A, and hypomethylation of the *HNF1A* gene promoter has been demonstrated in mature hepatocytes and renal tubular cells [29]. In kidneys, HNF1A regulates the expression of several organic anion transporters but its role in aquaporin or aquaglyceroporin expression has not been reported previously [29]. Furthermore, HNF1A level has been shown to be associated with differentiation of hepatocellular carcinoma (HCC) [28]. Given that AQP9 expression may be associated with differentiation of myeloid cells [14], it remains to be determined if HNF1A is also involved in myeloid differentiation. Finally, chronic exposure of the HCC cell line HepG2 to sub-toxic levels of arsenic leading to arsenic resistance has been shown to be associated with down-regulation of HNF1A expression [30]. This is in agreement with our result, in that decreasing HNF1A level would result in down-regulation of AQP9, which led to decreased arsenic entry into cells, thereby contributing to arsenic resistance.

## Conclusions

In conclusion, we have provided proof-of-principle evidence that a novel strategy of pharmacological up-regulation of AQP9 might be an approach to increase arsenic sensitivity in neoplastic cells. Furthermore, given that azacytidine is a proven treatment for AML, azacytidine, and As_2_O_3_ would be a logical drug combination to be further evaluated for the treatment of AML in future clinical trials.

## Additional file

Additional file 1: **Supplementary Information.** Supplementary materials and methods.
