# Supplementary material for: Azacytidine sensitizes acute myeloid leukemia cells to arsenic trioxide by up-regulating the arsenic transporter aquaglyceroporin 9
Source: J Hematol Oncol. 2015 May 8;8:46. doi: 10.1186/s13045-015-0143-3 (PMC4431177; doi:10.1186/s13045-015-0143-3)
Supplement: Supplementary file 1 — Supplementary Information. Supplementary materials and methods. [file 13045_2015_143_MOESM1_ESM.doc]

**Supplementary Information**

**Methods**

**Sequences of primers and siRNA, and reaction conditions**

1. **Reverse transcription polymerase chain reaction for gene quantification**

Target genes

***AQP9* primers**

Forward: 5′ AGT TGT TGG GAG CCT TTG TG 3′

Reverse: 5′ GTT CGC CAG AGA TAG ATA CGG AG 3′

***HNF1A* primers**

Forward: 5′ CCC CAG ATT CAG GAT CAG ACA 3′

Reverse: 5′ CCA TCA TGT TCC ATT TTT CGC 3′

Internal control gene

***GAPDH* primers**

Forward: 5′ GAA GGT GAA GGT CGG AGT CA 3′

Reverse: 5′ CTT CTA CCA CTA CCC TAA AG 3′

Polymerase chain reaction conditions

Initial denaturation: 95°C for 2 minutes

Cycling conditions: 40 cycles of 95°C for 5 seconds and 60°C for 1 minute.

1. **siRNA sequence for knockdown experiments**

**siRNA targeting *HNF1A***

5′ GTT TAT AGG GTT TGG TTA GTG G 3′

5′ GU AAU UCA UUG CCU CUG CUU CAA-3′

1. **Methylation specific polymerase chain reaction of *HNF1A* promoter**

**Primers for methylated DNA**

Forward: 5′ TGT CGG TCG GTA GGT AAA CG 3′

Reverse: 5′ CTT CCC CAT CGT CGT CCG 3′

**Primers for unmethylated DNA**

Forward: 5′ TGT TGG TTG GTA GGT AAA TGT AAT 3′

Reverse: 5′ AAT CTT CCC CAT CAT CAT CCA 3′

MSP conditions

Initial denaturation: 95°C for 2 minutes

For methyalted primers:

Cycling conditions: 30-40 cycles of 95°C for 30 seconds and 61°C for 1 minute.

For ummethyalted primers:

Cycling conditions: 30-40 cycles of 95°C for 30 seconds and 57°C for 1 minute.

1. **Combined bisulfite restriction analysis (COBRA) of *HNF1A* promoter**

Forward: 5′ GTT TAT AGG GTT TGG TTA GTG G 3′

Reverse: 5′ CCT TAC TAC AAA AAA ATC TCC AC 3′

PCR conditions

Initial denaturation: 95°C for 2 minutes

Cycling conditions: 40 cycles of 95°C for 30 seconds and 50°C for 1 minute.
